# Recent Researches on the Development of the Teeth

**Published:** 1858-10

**Authors:** 


### EDITORIAL DEPARTMENT.

*Recent Researches on the Development of the Teeth.*—M. Natalis Guillot has presented to the French Academy of Sciences, a memoir on the subject of the development of the teeth. He professes to have discovered in the jaws of embryos a peculiar temporary organ, within which the teeth are formed, and around which the bones of the jaws are built up. Having subserved this two-fold purpose, it disappears.

As might be expected, it changes its aspect at different stages of embryonic growth. At first it is made up of molecules or irregular and nucleated cells. At this time, the ivory and enamel, still soft, are formed. After this, it becomes fibrous by the elongation of its constituent molecules, and at this time the dental sac and the cement are formed. In the sheep, the first traces of the teeth appear in the midst of this organ, before the end of the first month of embryonic life, before muscles, nerves or blood-vessels can be distinguished upon the face, and even when the first traces of the bones are hardly to be met with. These primordial indications of teeth resemble little spheres formed by a multitude of molecules or cells. Those which are most easily discovered belong to the first dentition; about the third or fourth month, the germs of the second teeth are indistinctly seen in the human embryo. No envelop or sac belongs to these spheroids in this primitive condition.

Three divisions take place before solidification. One is central, destined to produce the dentine,—the second forms the enamel,—the outermost gives rise ultimately to the dental sac and produces the cement. This formation of the dental sac is really due only to the conversion of the formative organ into a fibrous substance, passing gradually into a tissue closely resembling periosteum. These phenomena occur during the formation of both sets of teeth.

Each of the initial spheroids of the teeth seems from the beginning to be a centre, about which the bony substance incessantly accumulates, till it forms a solid capsule more or less complete, in the interior of which is the sac of each tooth. This bony capsule, widely opened above the crown of the temporary and of the permanent molars, is, on the contrary, closed above the crowns of the teeth of replacement, in which a narrow channel, instead of a large opening, shows the excessive accumulation of bony matter. The teeth of replacement could never emerge from the capsules which so narrowly enclose them, unless a part of the wall forming these capsules disappeared to allow the emergence of the crown. This disappearance, due to an absorption of the bony molecules, is equally appreciable in the bony capsules which fix the teeth of the first dentition. It brings about the shedding of these organs.

While in several regions the jaws decrease and lose the substance of which they are composed; in other parts, these same jaws increase, extend and make room for the growing teeth. There is, therefore, in the bones of the jaws, a double series of molecular movements; in one of which the bones are partially atrophied and disappear entirely; while in the other, they grow at other points. This double movement is constantly going on in all the regions of the jaws during the development of the teeth; the effects being discoverable up to adult age. In consequence of the orderly sequence of these changes, the different diameters of the maxillary bones change constantly and regularly till the permanent dimensions of the face are attained.

The committee appointed to investigate this paper consisted of M. M. Flourens, Coste and J. Cloquet. We have not as yet seen their report.

Meanwhile, Dr. Magitot, as we learn from a review in *L'Art Dentaire*, has written an inaugural thesis on the development and structure of the human teeth. We shall briefly recapitulate the principal points of this thesis.

He adopts Goodsir's views in reference to the dental follicle as an involution of the mucous membrane, though, in consequence of the dissent of Purkinje and other observers, he thinks further researches are necessary, and promises to devote himself to this inquiry at some future period. He considers the intimate adherence between the gum and follicle from the third month of fœtal life to be incontestibly proved.

As for the structure of the follicle, he considers it to be composed of three distinct parts: the *wall* or *envelop;* the *dental pulp*, germs or papilla, the germ of the dentine; and the germ or organ of the enamel. The first of these differs in structure at different stages of development.

Before the commencement of dentinification it is a mere mass of fibro-cellular structure loosely grouped about the pulp. When the other parts have reached sufficient size to fill the sac, it is easily separated into two membranes, having each a distinct structure and function. The external envelop, a whitish, opaque membrane, forms a complete sac, adherent on one side to the inner surface of the gum, and extending on the other along the vascular and nervous pedicle which traverses it to join the pulp. Composed of a cellular web which resembles that of mucous membrane, of which it looks like a fold, furnished with a great number of capillaries, communicating with the vessels of the buccal mucous membrane, it lines by its outer surface the wall of the alveolus, and after the complete development of the tooth, extends along the root to form the alveolo-dental periosteum. The internal envelop, smooth and polished, gives attachment by two opposite points, to the two germs of the enamel and the dentine. This membrane has only a temporary existence subordinate to that of the germ of the enamel. After development it is confounded with the external membrane.

The *dental germ*, before the fourth month, is not bigger than the head of a pin, and is only a mass of finely granular amorphous matter, containing a great quantity of fibro-plastic ovoid nuclei; rarely presenting prolongations which, formed at the expense of their substance, appear at the fourth month on opposite points; the nuclei then become fusiform, new prolongations are produced at different points of the circumference of the nucleus which is soon surrounded by very numerous rays that ramify and form the cellular net-work of the pulp. When this lamellar evolution (*evolution lamineuse*) of the fibro-plastic nucleus is completed, the nucleus is atrophied and disappears at the same time that new nuclei originate in the midst of the organ, to undergo, in their turn the same evolution, till the constitution appears complete, which occurs about the fifth month of fœtal life.

As to the membrane called by Raschkow *membrana præformativa*, *basement membrane* (Bowman, Huxley,) *persistent capsule* (Nasmyth,) it does not exist in the follicle and cannot play a part in the formation of the dental elements. It appears to be only the superficial layer of the amorphous matter, which can be separated from the germ, sometimes, by a prolonged maceration, when it floats in the liquid in the form of very delicate membranous shreds. The nerves of the pulp terminate in free extremities and not by loops, the tips being conical or slightly swollen, so as to form a sort of button.

The *enamel germ*, according to our author, is the transparent, viscid mass which adheres to the inner surface of the wall of the sac and covers the germ of the dentine. The form of this gelatinous matter which appears in great quantity if the dental sac be carefully opened at the commencement of dentinification, varies with the form of the teeth. In relation by its external surface with the inner membrane of the sac to which it adheres and which supplies it with vessels, this organ corresponds by its inner surface to the salient portion of the bulb of the dentine on which it is moulded and of which it follows the contours and sinuosities. It descends upon the sides of this bulb without reaching its base. Thus the cavity of the follicle is seen to be filled with two organs inserted upon opposite portions of its walls and growing towards the centre. They meet and are moulded on one another, till by the formation of their respective products, enamel and dentine, they are separated. Then the enamel organ, whose functions are only temporary, is atrophied, while that of the dentine remains during life. While the summit of the dentine germ is being covered with dentine cells, the corresponding surface of the enamel organ is forming cells of enamel, which interlace with the first layers of dentine and are transformed into columns or prisms of enamel.

The enamel organ is therefore composed of two parts, the gelatinous mass and the cells of enamel, contrary to the assertions of Owen, Nasmyth, Todd and Bowman, this organ is provided with a capillary network, not so rich as that of the dentine germ, coming from the vessels of the internal membrane of the follicle.

Our author has not yet been able to find nervous filaments, nor has any one else demonstrated them.

The primary organs having been thus described, the *development* of the teeth is next considered. The first range of cells formed in the thickness of the amorphous matter surrounding the bulb, raises the superficial layer (*membrana præformativa*) which is afterwards atrophied. At this time, each cell presents the features just described, but changes soon take place, the commencement of which is marked by the disappearance of the nucleus which is completely atrophied. The cells become prismatic and undergo calcification, which makes of the soft cell a hard, brittle mass of dentine, the granulations disappear; the whole range of cells simultaneously transformed, presents a homogeneous aspect and a new layer of cells is produced beneath the first, to undergo the same modification. In reference to the production of the canaliculi, the author adopts the opinion of Schwann, Raschkow and

Huxley, which supposes them to be the intervals between the cells. The globules of dentine he supposes to be formed by an irregular accumulation of calcareous particles amid the dentinal cells, a sort of exaggeration of the organic action, of which the pulp had already afforded examples in the occurrences of crystals of hæmatoidin and masses of phosphates.

Of the various theories advanced to account for the development of dentine, our author adopts, with some modifications, that of *deposition*, which assumes that the ivory and enamel are produced by special elements, foreign to the germs that give rise to them. He supposes that the anatomical elements of the tooth are spontaneously developed on the surface of the germs, without the direct participation of their tissue, an action which he and his master, Charles Robin, call *autogeny*.

The development of the *enamel* proceeds after the following manner: After the deposition of the cells already described on the surface of the dentine, they press against one another, and assume a regularly prismatic form. Calcification taking place, the nucleus disappears, and the same process goes on in every successive layer that is deposited.

The *cement* is probably the result of the transformation of a special organ, underlying the follicular envelop, though further inquiries are necessary to establish the point. Among some of the pachydermata, such an organ is very apparent, but in man it is not easily made out.

In treating of the *structure* of the teeth, our author describes the dentine as composed of a fundamental substance, which is very finely granular and preserves no trace of the original structure of the dentine cells. The canaliculi he believes to be devoid of proper walls and to represent merely the intervals left between the primitive cells. During life they contain a transparent, colorless fluid, designed probably to facilitate the organic changes of this tissue which is alike destitute of vessels and nerves. He is disposed also to attribute a certain amount of organic change even to the enamel. The cement he conceives to be constantly growing like all other bony tissue.

In the description of the soft parts of the teeth we find nothing new.

